# Combination Therapy with Ciprofloxacin and Pentamidine against Multidrug-Resistant *Pseudomonas aeruginosa*: Assessment of In Vitro and In Vivo Efficacy and the Role of Resistance–Nodulation–Division (RND) Efflux Pumps

**DOI:** 10.3390/antibiotics12081236

**Published:** 2023-07-26

**Authors:** Megan Fletcher, Alex McCormack, Benjamin J. Parcell, Peter J. Coote

**Affiliations:** 1 Biomedical Sciences Research Complex, School of Biology, University of St Andrews, The North Haugh, St Andrews, Fife KY16 9ST, UK; mf616@exeter.ac.uk (M.F.); am572@st-andrews.ac.uk (A.M.); 2 NHS Tayside, Medical Microbiology, Ninewells Hospital and Medical School, Dundee DD1 9SY, UK; benjamin.parcell@nhs.scot

**Keywords:** *Galleria mellonella*, drug repurposing, antibiotic resistance, synergy, efflux pump inhibitor, MexAB-OprM, antibiotic resistance breaker

## Abstract

The aim of this work was to (i) evaluate the efficacy of a combination treatment of pentamidine with ciprofloxacin against *Galleria mellonella* larvae infected with an MDR strain of *P. aeruginosa* and (ii) determine if pentamidine acts as an efflux-pump inhibitor. Resistant clinical isolates, mutant strains overexpressing one of three RND efflux pumps (MexAB-OprM, MexCD-OprJ, and MexEF-OprN), and a strain with the same three pumps deleted were used. MIC assays confirmed that the clinical isolates and the mutants overexpressing efflux pumps were resistant to ciprofloxacin and pentamidine. The deletion of the three efflux pumps induced sensitivity to both compounds. Exposure to pentamidine and ciprofloxacin in combination resulted in the synergistic inhibition of all resistant strains in vitro, but no synergy was observed versus the efflux-pump deletion strain. The treatment of infected *G. mellonella* larvae with the combination of pentamidine and ciprofloxacin resulted in enhanced efficacy compared with the monotherapies and significantly reduced the number of proliferating bacteria. Our measurement of efflux activity from cells revealed that pentamidine had a specific inhibitory effect on the MexCD-OprJ and MexEF-OprN efflux pumps. However, the efflux activity and membrane permeability assays revealed that pentamidine also disrupted the membrane of all cells. In conclusion, pentamidine does possess some efflux-pump inhibitory activity, in addition to a more general disruptive effect on membrane integrity that accounts for its ability to potentiate ciprofloxacin activity. Notably, the enhanced efficacy of combination therapy with pentamidine and ciprofloxacin versus MDR *P. aeruginosa* strains in vivo merits further investigation into its potential to treat infections via this pathogen in patients.

## 1. Introduction

*Pseudomonas aeruginosa* is a Gram-negative opportunistic pathogen and a leading cause of mortality and morbidity among immunocompromised patients and those with cystic fibrosis (CF). The World Health Organization (WHO) has designated multidrug-resistant (MDR) *P. aeruginosa* as a critical pathogen requiring the development of new antibiotic classes because it poses a threat to human health [1]. The UK Health Security Agency notes that the 2020–2021 case fatality rates (number of deaths as a percentage of reported cases within 30 days of infection) for hospital-onset and community-onset cases of bacteremia were 33.9% and 23.8%, respectively [2]. *P. aeruginosa* infections range from minor skin infections to pneumonia and more serious infections of the bloodstream and urinary tract with immunocompromised patients, those undergoing invasive procedures, or those on prolonged antibiotic treatment being at highest risk [3,4]. *P. aeruginosa* is most common in patients being treated in the intensive care unit and causes 10–11% of nosocomial infections [5]. For ventilator-associated pneumonia and catheter-associated and central-line-associated infections, the incidence of MDR ranged from 18 to 20% in the United States from 2011 to 2014 [6]. *P. aeruginosa* is also a leading factor in disease progression for people with CF [7].

Antibiotic-resistant *P. aeruginosa* infections are often treated with dual combinations of antibiotics, usually consisting of a β-lactam with either an aminoglycoside or fluoroquinolone. The rationale behind this is that the simultaneous administration of two antibiotics with different modes of action increases the likelihood that the pathogen will be inhibited by at least one of the component drugs [8]. Receiving inappropriate initial antibiotic therapy correlates with patient mortality [9], meaning that combination therapy can potentially improve the efficacy of the initial therapy. However, definitive evidence that antibiotic combination therapy does result in enhanced efficacy is conflicting. Recently, Babich et al. [10] concluded, from a multicenter retrospective study, that the current antibiotics used in combination therapy resulted in no mortality advantage over monotherapy for *P. aeruginosa* bacteremia. The lack of new treatment options for MDR *P. aeruginosa* infections means that the ‘repurposing’ of already approved drugs, whose primary use is not as antibacterials, as antibiotics could represent a novel approach, particularly if these compounds are administered in combination with existing antibiotics [11,12].

A drug that could be ‘repurposed’ as an antibiotic adjuvant is the antiprotozoal drug pentamidine, which is used to treat trypanosomiasis and leishmaniasis. Pentamidine does possess weak antibacterial activity alone but has the most promise when used in combination with other drugs. In vitro, pentamidine was synergistic with rifampicin, erythromycin, and novobiocin against a range of Enterobacterales [13]. Against a suite of clinical MDR *P. aeruginosa* strains, pentamidine in combination with imipenem, meropenem, ciprofloxacin, and levofloxacin showed synergistic inhibition of some of the strains in both checkerboard and time-kill assays [14]. In two in vivo studies, a combination of pentamidine and novobiocin was shown to protect mice with a systemic infection of colistin-resistant *Acinetobacter baumannii* [13,15]. To date, no studies have assessed the potential of combination treatments of pentamidine with antibiotics against MDR *P. aeruginosa* infections in vivo.

The mechanism underpinning the synergistic inhibition of bacteria that occurs when pentamidine and antibiotics are used in combination has been attributed to pentamidine binding to the lipopolysaccharide (LPS) in the outer membrane of Gram-negative bacteria and inducing enhanced permeability and, thus, improved access of the co-administered antibiotic into the cells [13]. The interaction of pentamidine with the outer membrane and LPS of Gram-negative bacteria led us to hypothesize that an alternative mechanism explaining the synergy of pentamidine with a host of different antibiotics could be due to a direct or indirect inhibition of membrane-bound efflux-pumps.

Efflux-pumps contribute to reduced efficacy of several important classes of antibiotics used against *P. aeruginosa* and contribute to resistance [16]. In *P. aeruginosa,* the members of the resistance–nodulation–division (RND) family of efflux-pumps are the most significant mediators of antibiotic resistance. The RND family has twelve members but four are closely associated with antibiotic efflux: MexAB-OprM, MexXY-OprM, MexCD-OprJ, and MexEF-OprN [17]. These four pumps transport an overlapping range of antibiotic substrates, in particular, the fluoroquinolones. MexAB-OprM is the most important efflux pump that mediates antibiotic resistance because it is constitutively expressed and, thus, confers intrinsic resistance on *P. aeruginosa* [17]. Moreover, the deletion of *mexA* or *oprM* results in hyper-susceptibility to antibiotics [18]. Notably, it can extrude a broad range of substrates and can efflux almost all classes of antibiotics. In contrast, the expression of MexCD-OprJ and MexEF-OprN requires exposure to a range of compounds or environmental stimuli [19]. The substrate range of MexCD-OprJ is similar to that of MexAB-OprM, while MexEF-OprN has a narrower range [17]. The deletion of components of either of these two pumps does not affect antibiotic susceptibility [20]. Mutations in efflux-pump regulatory genes can result in the overexpression of any of these pumps and confer a MDR phenotype. Importantly, these mutations are commonly identified in clinical isolates; for example, mutations in *nalB* [21], *nfxB* [22], and *nfxC* [23] result in overexpression of MexAB-OprM, MexCD-OprJ, and MexEF-OprN, respectively.

The aim of this study was two-fold. Firstly, it aimed to identify if pentamidine in combination with the fluoroquinolone, ciprofloxacin, could overcome resistance to the drug, act as a ‘resistance-breaker’, and result in enhanced efficacy in vivo against infections with ciprofloxacin-resistant *P. aeruginosa* strains in a *Galleria mellonella* larva (Greater Wax moth) infection model. Secondly, it aimed to determine if pentamidine had any inhibitory effect on the activity of RND efflux pumps that could possibly account for the synergistic inhibition observed when the compound is used in combination with antibiotics. To test this hypothesis, four characterized *P. aeruginosa* strains were used, three of which overexpress individual RND efflux pumps (MexAB-OprM, MexCD-OprJ, and MexEF-OprN [24]), and one strain with all three of the pumps deleted.

## 2. Results

### 2.1. Sensitivity of P. aeruginosa Strains to Ciprofloxacin and Pentamidine

The strains used in this study are shown in Table 1. According to the European Committee on Antimicrobial Susceptibility Testing (EUCAST), sensitivity to ciprofloxacin is defined as ≤0.001 mg/L and resistance > 0.5 mg/L [25]. The MICs for the *P. aeruginosa* strain used in this study are shown in Table 2. The most resistant were NCTC13437 and the clinical isolate CR-BJP-VIM, but the three strains overexpressing certain RND efflux pumps were also resistant. NCTC13437 is known to be resistant to fluoroquinolones by an unknown mechanism [26]. The wild-type control strain for the efflux-pump mutant strains (PAM1020) had normal susceptibility, and the strain with three RND efflux pumps deleted was most susceptible. 

Only PAM1626, with three RND efflux pumps deleted, was susceptible to pentamidine, implying that functioning RND efflux pumps mediate tolerance to this drug. All the other strains had MICs of 256 mg/L or greater, indicating that pentamidine has minimal inhibitory activity against *P. aeruginosa*. 

### 2.2. Exposure of P. aeruginosa Strains to Combinations of Ciprofloxacin and Pentamidine In Vitro Results in Synergistic Bactericidal Inhibition

**Figure 1:**
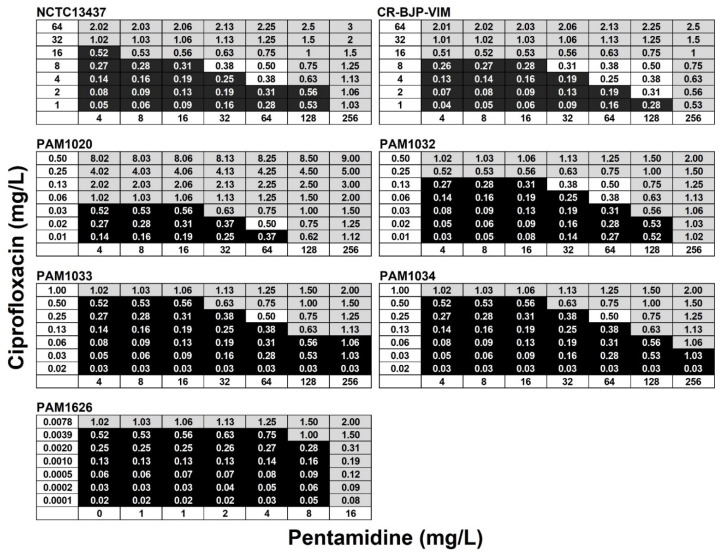
The effect of combination of ciprofloxacin with pentamidine on the growth of *P. aeruginosa* strains in vitro. Fractional inhibitory concentration indices (FICIs) of ciprofloxacin combined with pentamidine were calculated versus each strain after 24 h in MHB at 37 °C. Black squares indicate FICI values where bacterial growth occurred. Gray squares indicate wells where the FICI values were ≥0.5 (indicating inhibition was not synergistic). White squares show FICI values of 0.5 or less where bacterial growth was inhibited and, thus, indicate synergistic inhibition of growth. The experiment was performed in duplicate, and a representative result is shown.

Checkerboard assays showing the effect of different pentamidine and ciprofloxacin combinations on the growth of all the *P. aeruginosa* strains are shown in Figure 1. The strongest synergies observed were against the clinical isolate CR-BJP-VIM (FICI—0.25), NCTC13437 (FICI—0.38), and PAM1032, overexpressing the MexAB-OprM efflux pump (FICI—0.38). Weaker synergy (FICI—0.5) was observed against both PAM1033 and PAM1034, overexpressing the MexCD-OprJ and MexEF-OprN pumps, respectively. Notably, no synergistic inhibition of PAM1626 was detected.

**Figure 2:**
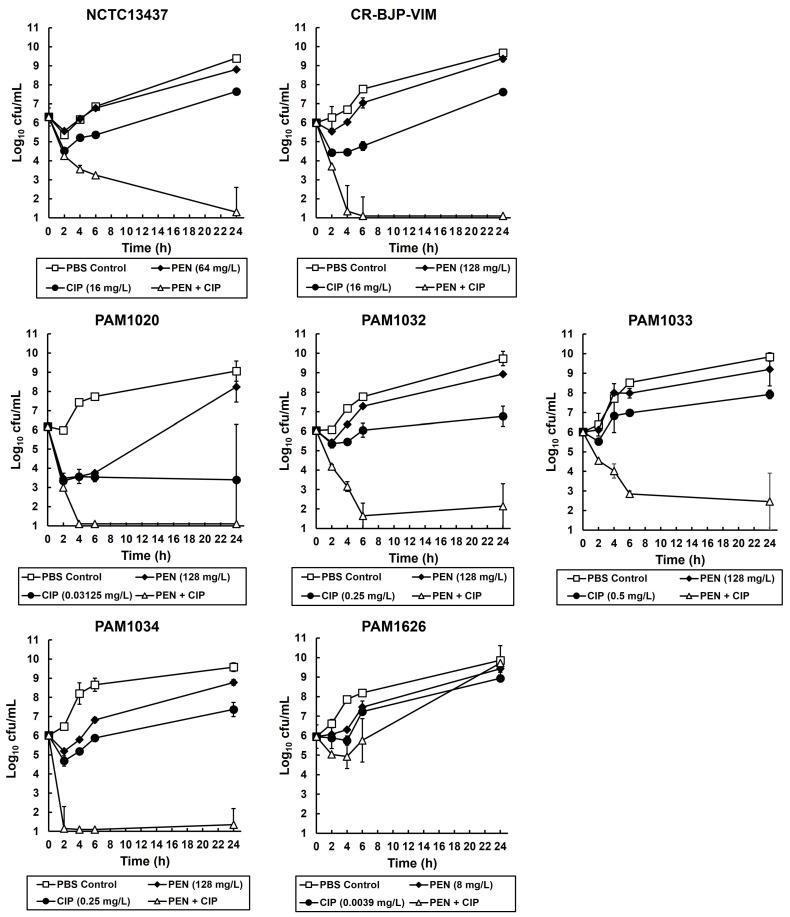
Time-kill assays comparing the effect of exposure to ciprofloxacin and pentamidine alone with the drugs in combination on the growth and viability of *P. aeruginosa* strains in vitro. Bacteria were exposed to ciprofloxacin or pentamidine concentrations at either MIC_0.25_ or MIC_0.5_ for 24 h at 37 °C in MHB. Concentrations of the individual drugs used alone or in combination are indicated in the figure legend. For each condition tested, viable bacteria were measured after 2, 4, 6, and 24 h of exposure. Each experiment was performed in duplicate, and the mean ± SEM is shown.

To determine if the synergistic growth inhibition observed in the checkerboard assays was bacteriostatic or bactericidal, time-kill assays were performed on all the *P. aeruginosa* strains. The effect of exposure to the single drugs (at MIC_50_ or MIC_25_) and the combination (also at MIC_50_ or MIC_25_ for each drug) on the viability of each strain after 24 h of exposure at 37 °C is shown in Figure 2. Control populations of all strains, mock treated with PBS, increased in cell number over the duration of the experiment. Exposure to pentamidine alone resulted in an initial minor loss in viability of all strains after 2 h of exposure, but after 24 h, all strains recovered, and population viabilities were similar to those of the PBS controls. Similarly, exposure to ciprofloxacin alone also resulted in an initial loss in viability of all strains after 2 h of exposure, but after 24 h, all strains still recovered (except for PAM1020), and viability had increased, albeit to a lower cell number than the PBS controls. The combination of pentamidine with ciprofloxacin resulted in a steady loss of viability after 6 h of exposure with all strains except PAM1626, the strain with three RND efflux pumps deleted. Notably, in the strains where the combination treatment reduced viable numbers after 6 h, no recovery of any of the populations was observed after 24 h of exposure, and viable numbers either declined further or remained static. The American Society for Microbiology (ASM) definition of synergy with time-kill assays is a ≥2-log_10_ decrease in cfu/mL between the combination and its most active constituent after 24 h, and the number of surviving organisms in the presence of the combination must be ≥2 log_10_ cfu/mL below the starting inoculum (https://journals.asm.org/abbreviations-conventions, accessed on 28 June 2023). By this definition, the inhibition of *P. aeruginosa* by the combination of pentamidine with ciprofloxacin is synergistic, and these results support the results observed in the checkerboard assays. Despite being bactericidal, the combination did not eliminate all bacteria over the duration of the experiment.

Notably, in both the checkerboard and time-kill assay, the combination did not result in the synergistic inhibition of PAM1626, the strain with the triple deletion of RND pumps. This implies that a potential target of the synergistic combination of pentamidine with ciprofloxacin could be RND efflux pumps.

### 2.3. Survival of G. mellonella Larvae Infected with Different P. aeruginosa Strains and Treated with Ciprofloxacin or Pentamidine Alone Correlates with the In Vitro MIC Values

**Figure 3:**
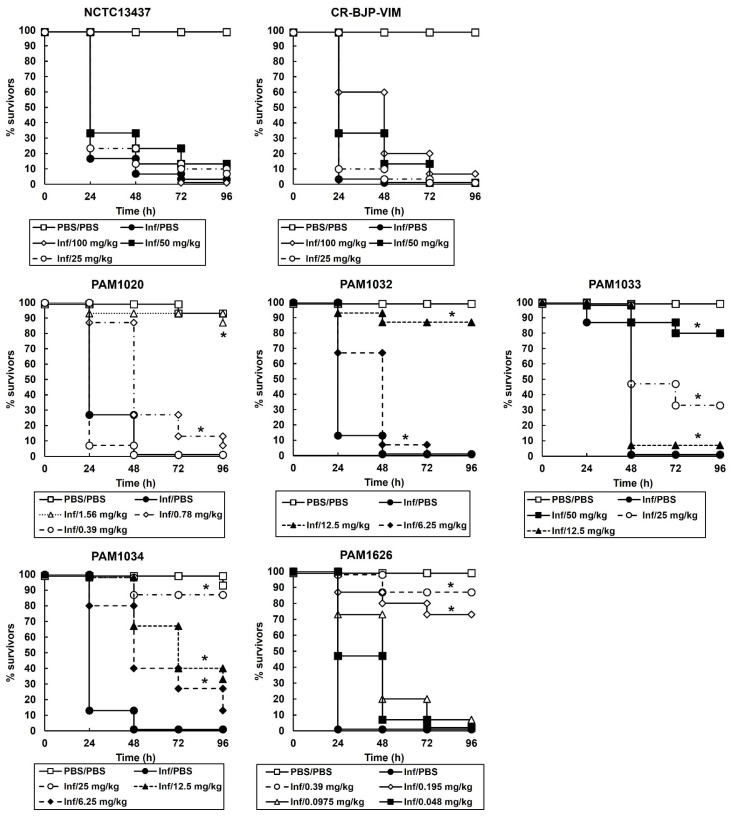
Effect of treatment with ciprofloxacin monotherapy on survival of *G. mellonella* larvae infected with 2.5 × 10^3^ cfu/mL of *P. aeruginosa* strains. Infected larvae were treated with either PBS (mock ‘treated’) or ciprofloxacin at a range of doses, as indicated in the figure legends for larvae infected with each strain and incubated at 37 °C. A single dose of the antibiotic treatments was administered 2 h p.i. Surviving larvae were counted every 24 h for 96 h. The uninfected PBS/PBS group represents larvae sham-infected with sterile PBS and treated with sterile PBS. * Significantly enhanced survival compared to infected larvae treated with PBS (*p* < 0.05, log rank test with Holm correction for multiple comparisons); *n* = 30 (pooled from duplicate experiments).

Initial experiments determined the efficacy of monotherapy (a single dose of either ciprofloxacin (Figure 3) or pentamidine administered 2 h post-infection (p.i) on *G. mellonella* larvae infected with a lethal dose (2.5 × 10^3^ cells/mL) of each of the *P. aeruginosa* strains. The data for pentamidine treatment alone is not shown because even at the highest dose tested (100 mg/kg), no therapeutic benefit was observed for larvae infected with any of the strains except PAM1626 where 20% of larvae survived 96 h p.i. These experiments identify the doses of ciprofloxacin and pentamidine that provide minimal therapeutic benefit that can then be applied in combination treatments and allow the ready detection of any enhanced efficacy of combination therapy over monotherapy. 

A single dose of ciprofloxacin given to larvae infected with either *P. aeruginosa* NCTC13437 or the clinical isolate CR-BJP-VIM offered no therapeutic benefit after 96 h incubation at 37 °C at the highest dose administered (100 mg/kg). 

For the remaining *P. aeruginosa* strains, where treatment with a single dose of ciprofloxacin conferred a therapeutic benefit to infected larvae, treatment with ciprofloxacin resulted in dose-dependent efficacy. However, the dose of the antibiotic required to do this was strain dependent. For example, infection with the strains overexpressing individual RND efflux pumps required higher doses of ciprofloxacin to confer therapeutic benefit than the parent strain, PAM1020, or the strain with the triple RND efflux-pumps deletions, PAM1626. In fact, the order of resistance to ciprofloxacin treatment of larvae infected with each of the *P. aeruginosa* strains was (most resistant first) NCTC13437 > CR-BJP-VIM > PAM1033 > PAM1034 > PAM1032 > PAM1020 > PAM1626. This efficacy of ciprofloxacin in vivo closely correlated with the MIC values determined in vitro (Table 2) and indicates a significant role for the different RND efflux pumps that are overexpressed (or deleted) in these strains in conferring varying degrees of resistance to ciprofloxacin.

In contrast, treatment with a single dose of pentamidine at the highest dose tested (100 mg/kg) resulted in no therapeutic benefit to infected larvae after 96 h of incubation at 37 °C for all the *P. aeruginosa* strains, except PAM1626, where 20% of larvae survived after treatment with the highest dose tested of 100 mg/kg. This lack of efficacy of pentamidine corroborated the very high MIC values that were observed for this compound (apart from that observed for PAM1626 (Table 2)) and confirmed that the efficacy of pentamidine in vivo, like ciprofloxacin, also correlated with the MIC values measured in vitro.

### 2.4. Combination Therapy of Ciprofloxacin with Pentamidine of G. mellonella Larvae Infected with P. aeruginosa Results in Enhanced Efficacy Compared to Monotherapies

**Figure 4:**
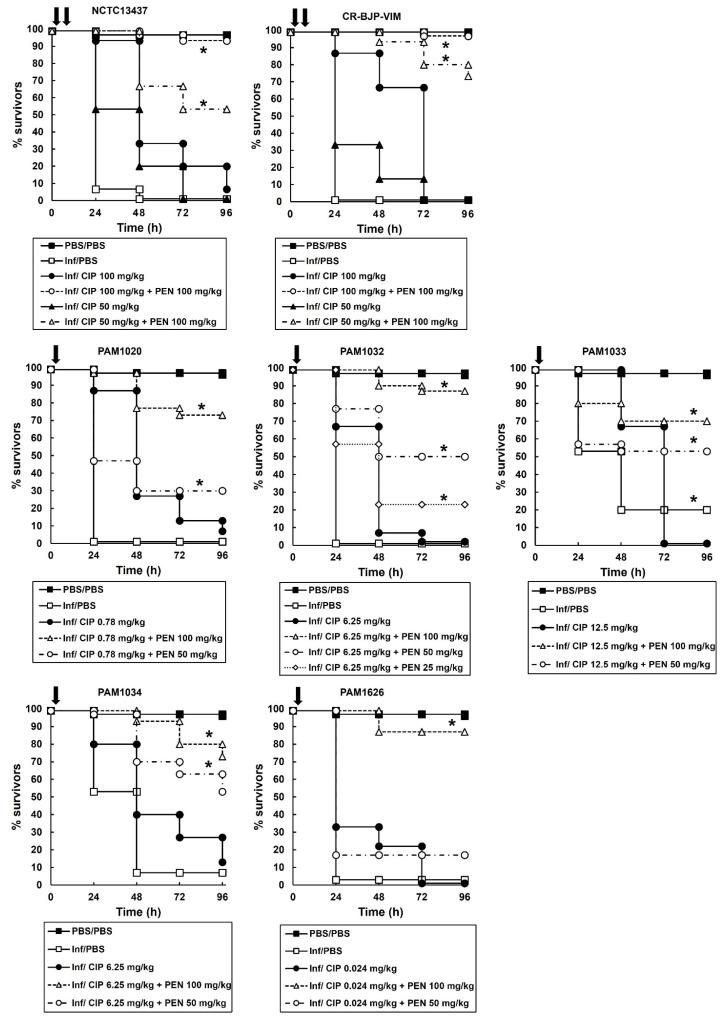
Effect of treatment with ciprofloxacin monotherapy or ciprofloxacin with pentamidine combinations, on survival of *G. mellonella* larvae infected with 2.5 × 10^3^ cfu/mL of *P. aeruginosa* strains. Infected larvae were treated with PBS (mock ‘treated’), ciprofloxacin monotherapies, or ciprofloxacin with pentamidine combinations at the doses indicated in the figure legends for each strain. Either a single (indicated by one vertical black arrow on the graph) or double (indicated by two vertical black arrows on the graph) dose of the treatments was administered 2 h or 2 and 4 h p.i, respectively. Larvae were incubated at 37 °C, and surviving larvae were counted every 24 h for 96 h. The uninfected PBS/PBS group represents larvae sham-infected with sterile PBS and treated with sterile PBS. * Significantly enhanced survival compared to each monotherapy alone (*p* < 0.05, log rank test with Holm correction for multiple comparisons); *n* = 30 (pooled from duplicate experiments).

Based on the data obtained from monotherapies of ciprofloxacin or pentamidine, doses of each individual drug that resulted in minimal therapeutic benefit were selected for testing in combination. The effect of combination treatment compared with monotherapies is shown in Figure 4. *P. aeruginosa* strains were treated with a single dose at 2 h p.i (PAM strains) or a double dose at 2 and 4 h p.i (NCTC13437 and CR-BJP-VIM). Two doses were required for the latter strains because a single dose resulted in only minor enhanced efficacy of the combination compared to the monotherapies. This is consistent with these two strains showing the greatest resistance to ciprofloxacin monotherapy in vitro (Table 2) and in vivo (Figure 3). With each *P. aeruginosa* strain, combination therapy with ciprofloxacin and pentamidine resulted in significantly enhanced efficacy compared to the sham treatment with PBS or each monotherapy (Figure 4). This enhanced efficacy is consistent with the synergy between ciprofloxacin and pentamidine that was shown in vitro (Figure 1 and Figure 2). It should be noted that the enhanced efficacy observed with PAM1626, where no synergy was observed in vitro, is due to the much lower MIC of pentamidine (Table 2) and the low efficacy of pentamidine treatment alone that was observed with this strain only (20% survival 96 h p.i after administration of a 100 mg/kg dose).

**Figure 5:**
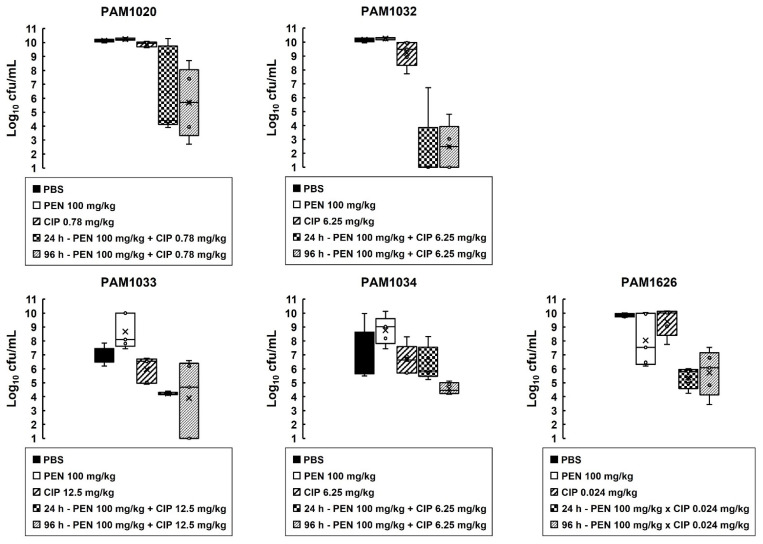
Box plots showing the effect of ciprofloxacin or pentamidine monotherapies or a combination of both drugs on the internal burden of *P. aeruginosa* strains in *G. mellonella* larvae. Larvae were infected with 2.5 × 10^3^ cfu/mL of *P. aeruginosa* strains and treated with either PBS (mock ‘treated’), a single dose of ciprofloxacin or pentamidine, a combination of both drugs, with the doses indicated in the figure legend for each strain at 2 h p.i. Larvae were incubated at 37 °C, and the internal burden of *P. aeruginosa* was determined from five individual larvae per treatment group after 24 and 96 h (for the combination treated larvae only) at 37 °C. The ‘x’ indicates the mean, the bar indicates the median, and the error bars show the highest and lowest values within the dataset. Outlier data are shown as independent points. For each strain, the combination treatment showed a significant reduction in bacterial burden compared with each monotherapy (*p* < 0.05, the Mann–Whitney U test; *n* = 5).

The effect of the combination treatment on the numbers of *P. aeruginosa* cells (PAM strains only) inside infected larvae is shown in Figure 5. With all infecting strains, the internal burden of *P. aeruginosa* within the larvae was reduced after combination therapy compared to sham treatment with PBS or each monotherapy (Figure 5). The effectiveness of the combination treatment in reducing the bacterial burden was strain dependent and correlated with the degree of resistance to ciprofloxacin shown in vitro (Table 2) and in vivo (Figure 4). Notably, after 96 h p.i, the reduction in bacterial burden compared to PBS or the monotherapies was maintained, but the infecting bacteria were never eliminated completely by the combination treatment. For example, with strain PAM1020, the combination treatment resulted in approximately 70% of infected larvae surviving at 96 h p.i, with an approximate 4.5-log_10_ reduction in bacterial numbers compared with monotherapies, but with a mean remaining bacterial burden of approximately 5-log_10_ cfu/mL. This trend of the survival of some infecting bacteria over the duration of the infection, despite the combination treatment resulting in enhanced survival of the larvae, was replicated with each of the *P. aeruginosa* strains (Figure 3).

### 2.5. Exposure to Pentamidine Disrupts the Activity of Specific RND Efflux Pumps in P. aeruginosa

**Figure 6:**
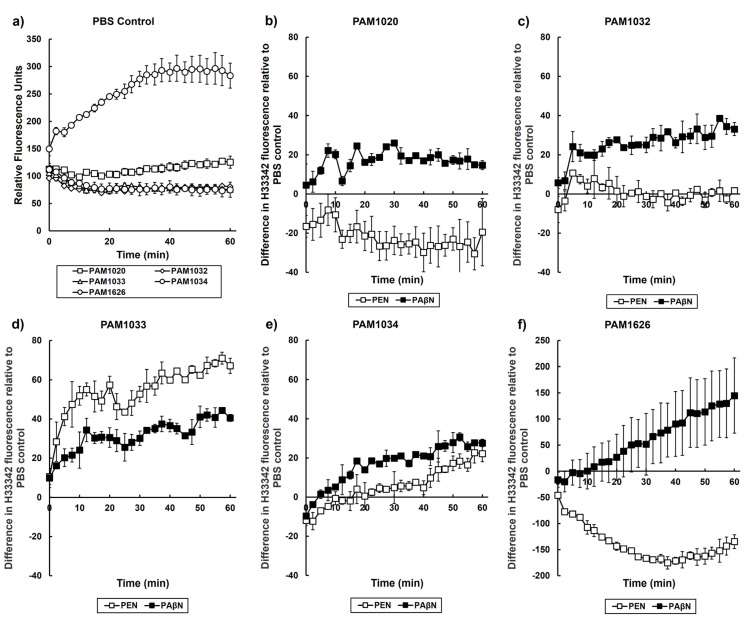
Hoechst 33,342 (H33342) fluorescence in the presence of *P. aeruginosa* strains exposed to pentamidine (PEN) (256 mg/L), phenylalanine-arginine β-naphthylamide (PAβN) (25 mg/L), or PBS at 37 °C. Fluorescence of H33342 for all strains in the presence of PBS is shown in (**a**). Change in H33342 fluorescence relative to PBS after exposure to pentamidine or PAβN is shown for each strain: (**b**) PAM1020, (**c**) PAM1032, (**d**) PAM1033, (**e**) PAM1034, and (**f**) PAM1626. Data shown are the mean of two experiments using independent replicates. Error bars indicate the ±standard error mean.

The antibacterial action of pentamidine has been attributed to the disruption of the outer membrane of Gram-negative bacteria via interaction with the lipopolysaccharide [13]. We reasoned that this could interfere with the function of the membrane-bound component of the RND efflux pumps and that pentamidine could result in efflux-pump inhibition. To determine if the basis of the synergy between ciprofloxacin and pentamidine was due to pentamidine acting as an efflux pump inhibitor, the retention and efflux of Hoechst 33342 (H33342) was measured. H33342 is a dye that can bind to the adenine-thymine regions of DNA, thus fluorescing only when it has entered a cell [27]. Therefore, accumulation assays of H33342 can determine whether the efflux is inhibited. H33342 assays were performed with the *P. aeruginosa* strains overexpressing certain RND pumps in the presence of PAβN (25 mg/L), a known efflux-pump inhibitor (EPI) at a concentration (25 mg/L) shown previously to inhibit RND efflux pumps [28], and pentamidine (256 mg/L) (Figure 6). 

In the control experiments, after exposure to PBS, the deletion of three RND efflux pumps resulted in a large net accumulation of H33342 within these cells compared to the other strains (Figure 6a). This is consistent with the loss of the efflux pumps allowing the dye to accumulate inside the cells and fluoresce due to a reduced rate of removal. Furthermore, the net accumulation of H33342 within the three strains overexpressing individual RND efflux pumps was reduced compared to the parent strain, PAM1020. Again, this is consistent with these three strains being capable of removing more H33342 from the inside of the cells compared to the parent due to enhanced pump activity (Figure 6a).

With each strain, exposure to the known EPI, PAβN, resulted in enhanced net accumulation of H33342 relative to that seen after exposure to PBS (Figure 6b–f). This is consistent with PAβN acting as an EPI and reducing the efflux of H33342 from inside the cells. Notably, exposure to pentamidine resulted in net accumulation of H33342 in strain PAM1033 (overexpressing MexCD-OprJ) (Figure 6d) and, to a lesser extent, PAM1034 (overexpressing MexEF-OprN (Figure 6e). With the parent strain, pentamidine resulted in a small net decrease in H33342 accumulation (Figure 6b), and with strain PAM1032 (overexpressing MexAB-OprM), there was no change in net accumulation of the dye relative to PBS (Figure 6c). In PAM1626, with three RND efflux pumps deleted, exposure to pentamidine resulted in a large net loss of H33342 from inside the cells, indicating a loss of integrity of the cell membrane, and this was consistent with the greater inhibitory action of pentamidine against this strain (Table 2).

### 2.6. Exposure to Pentamidine Disrupts the Outer Membrane of P. aeruginosa

**Figure 7:**
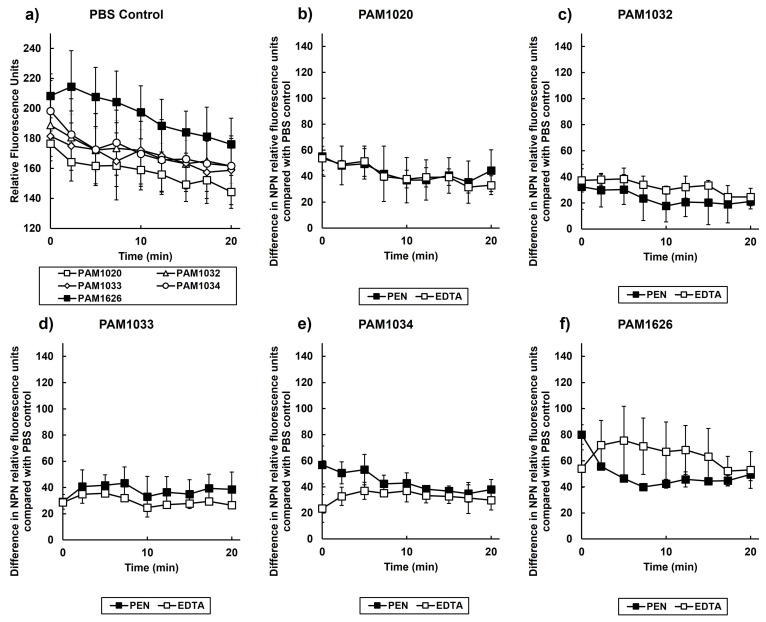
1-N-phenylnaphthylamine (NPN) fluorescence in the presence of *P. aeruginosa* strains exposed to pentamidine (PEN) (759 μM/256 mg/L) and ethylenediaminetetraacetic acid (EDTA) (100 μM). Fluorescence of NPN for all strains in the presence of PBS is shown in (**a**). Change in NPN fluorescence relative to PBS after exposure to pentamidine or EDTA is shown for each strain: (**b**) PAM1020, (**c**) PAM1032, (**d**) PAM1033, (**e**) PAM1034, and (**f**) PAM1626. Data shown are the mean of two experiments using independent replicates. Error bars indicate the ±standard error mean.

The previous results, which are supported by other studies [13], suggest that pentamidine is capable of binding to the membranes of Gram-negative bacteria. N-Phenyl-1-naphthylamine (NPN) assays were used to determine the extent of any permeabilizing effect pentamidine had on *P. aeruginosa* membranes. NPN fluorescence only occurs if the molecule binds to nonpolar environments, such as the cell membrane [29,30]. The effect of exposing *P. aeruginosa* to pentamidine on NPN fluorescence is shown in Figure 7.

As a positive control, the effect of exposure to a concentration of EDTA (100 µM) that is known to permeabilize the outer membrane of Gram-negative bacteria was also measured [31]. After exposure to PBS, there was no significant difference in NPN fluorescence between the parent strain and the three strains with overexpression of three RND pumps (Figure 7a). There was an indication of an increase in NPN fluorescence in the strain with three RND pumps deleted, but this was not significant (Figure 7a). These results show that the increased expression of RND pumps, or their deletion, had no significant effect on NPN fluorescence and, thus, membrane permeability.

In contrast, exposure of all the strains to pentamidine or EDTA resulted in increased NPN fluorescence (Figure 7b–f). With each strain, the increase in fluorescence induced by pentamidine at a concentration of (256 mg/L or 759 µM) was very similar to that induced by 100 µM EDTA. These results confirm that pentamidine does have a membrane-permeabilizing effect on *P. aeruginosa,* but it is less potent than EDTA. In addition, the degree of permeabilization induced by pentamidine appeared to be independent of the status of RND efflux-pump expression or presence. In conclusion, pentamidine does permeabilize *P. aeruginosa* membranes, and this effect is not influenced by RND efflux pumps. 

## 3. Discussion

In this work, the combination of the antiprotozoal drug pentamidine with ciprofloxacin showed synergistic, bactericidal inhibition of a group of fluoroquinolone-resistant strains of *P. aeruginosa*. This is supported by the earlier study by Herrera-Espejo et al. [14] that showed varying degrees of synergistic inhibition by pentamidine in combination with a range of antibiotics against a group of seven antibiotic-resistant *P. aeruginosa* clinical isolates. The authors observed the best synergy against six and five of the seven strains tested when pentamidine was combined with imipenem or meropenem, respectively. Pentamidine in combination with ciprofloxacin showed synergy against four out of the seven strains tested. This contrasts with the data presented here where the same combination inhibited all the *P. aeruginosa* strains tested in a synergistic fashion, except the strain with three RND efflux pumps deleted. 

Synergistic inhibition by combinations of pentamidine with antibiotics other than fluoroquinolones has also been shown versus a range of other Gram-negative pathogens, including *Acinetobacter baumannii*, *Klebsiella pneumoniae*, *Escherichia coli*, and *Enterobacter cloacae* [13,32]. Notably, prior to this study, only Stokes et al. [13] had shown that the in vitro synergy of pentamidine in combination with antibiotics translates into enhanced efficacy over antibiotic monotherapy in vivo—demonstrating that the combination of rifampicin or novobiocin with pentamidine protected mice infected with colistin-resistant *A. baumannii*. Thus, the results reported here, showing that a combination treatment of pentamidine with ciprofloxacin results in enhanced efficacy over monotherapy in *G. mellonella* larvae infected with MDR *P. aeruginosa* strains, provides additional evidence of the potential for using pentamidine as an antibiotic ‘resistance-breaker’ against MDR human pathogens.

An important consideration when repurposing pentamidine as a ‘resistance-breaker’ is the possible doses that can be administered to patients. In the UK, the British National Formulary (BNF: https://bnf.nice.org.uk/drugs/pentamidine-isetionate/, accessed on 29 June 2023) lists a nebulized inhalation dose of 300 mg every four weeks for prophylaxis of *Pneumocystis jirovecii* (*Pneumocystis carinii*) pneumonia and 4 mg/kg by injection for treatment of trypanosomiasis, or visceral leishmaniasis. These doses are lower than the effective dose used in combination with ciprofloxacin in this study, and it is known that pentamidine monotherapy does have toxic side effects, including nephrotoxicity [33]. Therefore, additional research optimizing the doses that can be employed in combinations with antibiotics would be essential. Notably, recent research has identified analogues of pentamidine with reduced toxicity that still retain their antibiotic potentiating effect versus mice infected with *Acinetobacter baumannii* [15].

The mechanism underpinning the synergistic interaction between pentamidine and antibiotics is proposed to be due to a membrane-disrupting effect of pentamidine on Gram-negative bacteria that results in the increased accumulation and, thus, potentiation, of antibiotic activity [13]. An alternative explanation could be that the pentamidine-induced membrane disruption results in the inhibition of efflux-pump activity that could also result in antibiotic potentiation. Four *P. aeruginosa* RND pumps (MexAB-OprM, MexCD-OprJ, MexEF-OprN, and MexXY-OprM) are known to efflux fluoroquinolones such as ciprofloxacin and contribute to resistance to this class of antibiotics [34]. In this study, the only strain for which synergistic inhibition between pentamidine and ciprofloxacin was not observed was *P. aeruginosa* PAM1626, with three RND efflux pumps deleted (*mexAB-oprM, mexCD-oprJ,* and *mexEF-oprN*; Figure 1 and Figure 2). This implies that the expression of these efflux pumps is necessary for synergistic inhibition to occur. The fact that only this strain was sensitive to pentamidine alone in vitro (Table 2) also implies that pentamidine does interact with the RND efflux pumps perhaps as a potential substrate. In contrast, the measurement of the accumulation of NPN in the outer membrane revealed that exposure to pentamidine (256 mg/L or 759 µM) resulted in an increase in membrane permeability that was equivalent to that induced by exposure to EDTA at a concentration (100 µM) known to permeabilize the outer membrane of Gram-negative bacteria [31]. The degree of membrane disruption induced by pentamidine was independent of the status of the three RND pumps because the overexpression of each pump individually or deletion of all three made no significant difference to the level of NPN fluorescence. Thus, these NPN data support the earlier conclusion of Stokes et al. [13] that pentamidine disrupts the outer membrane of Gram-negative bacteria. 

Despite pentamidine increasing membrane permeability, the measurement of the accumulation of H33342, an efflux pump substrate, revealed enhanced accumulation of H33342 only in the strains overexpressing MexCD-OprJ (Figure 6d) and, to a lesser extent, MexEF-OprN (Figure 6e). This implies that pentamidine has either specific inhibitory activity on these two RND efflux pumps or competes as a substrate with H33342. This effect was absent in the strain overexpressing MexAB-OprM. With the parent strain and the strain with three RND pumps deleted, exposure to pentamidine resulted in a net efflux of H33342 from the cells that was indicative of a non-specific membrane permeabilizing effect. Finally, it cannot be discounted that the mutations (*nalB*, *nfxB*, and *nfxC*) in the regulatory genes present in these strains that result in the overexpression of MexAB-OprM, MexCD-OprJ, and MexEF-OprN, respectively, could also have other unknown consequences that influence the relationship between outer membrane integrity and RND efflux pump activity.

In summary, these results suggest that pentamidine does possess some EPI activity that is specifically directed at the MexCD-OprJ pump, and to a lesser extent MexEF-OprN. However, the fact that pentamidine also resulted in a large net decrease in accumulation of H33342 dye in the strain with three RND pumps deleted and, to a lesser extent, in the parent strain also supports the previous findings that the drug also has a more general detrimental effect on membrane integrity. Regardless of the mechanism of synergy between pentamidine and ciprofloxacin, the finding that this combination can enhance the efficacy of the antibiotic in vivo and act as a ‘resistance-breaker’ versus resistant *P. aeruginosa* infections merits further investigation of its potential to treat infections by this pathogen in patients.

## 4. Materials and Methods

### 4.1. Bacteria and Growth Media

MDR isolates were defined using the Centers for Disease Control and Prevention (CDC) definition of an isolate that is resistant to at least one antibiotic in three or more drug classes. The *P. aeruginosa* strains used in this study are shown in Table 1. Strain PAO1 and the efflux pump mutants were a kind gift from Dr. Olga Lomovskaya, Qpex Biopharma, USA. *P. aeruginosa* NCTC13437 was obtained from the National Collection of Type Cultures (NCTC) (http://www.phe-culturecollections.org.uk/collections/nctc.jsp, accessed on 29 June 2023). Strain CR-BJP-VIM, an MDR *P. aeruginosa* clinical isolate that is resistant to gentamicin, ciprofloxacin, piperacillin–tazobactam, ceftazidime, meropenem, and imipenem, was positive for a VIM (Verona Integron-Mediated Metallo-β-lactamase) gene. This clinical strain was isolated from a leg ulcer swab and was provided by the co-author Dr. Benjamin Parcell, NHS Tayside. All strains were cultured overnight in Mueller–Hinton Broth (MHB; Merck, Darmstadt, Germany) at 37 °C, with shaking, to prepare inocula for drug susceptibility testing in vitro and efficacy testing in vivo. 

### 4.2. Reagents and G. mellonella Larvae

All chemicals were purchased from Sigma-Aldrich Ltd. (Dorset, UK). Stock solutions of ciprofloxacin (CIP), pentamidine (PEN), phenylalanine-arginine β-naphthylamide (PAβN), and Hoechst 33,342 (H33342) were prepared in sterile deionized water. A 5 mL stock solution (10 mg/L) of ciprofloxacin (CIP) was made up in water with 100 µL of 1 M HCl to fully dissolve. A stock solution of 10 mM 1-N-phenylnaphthylamine (NPN) was first prepared in 99% methanol and diluted to 40 µM in water for NPN assays. In this assay, the remaining methanol was diluted to 0.39% and had no effect on viability of *P. aeruginosa* cells. *G. mellonella* larvae were obtained from UK Waxworms Ltd. (Sheffield, UK).

### 4.3. Antibiotic Susceptibility and Checkerboard Assay

Minimum inhibitory concentrations (MICs) of PEN and CIP against the *P. aeruginosa* strains were determined in 96-well microplates, as previously described [35]. Briefly, doubling dilutions of antimicrobials were prepared in MHB and subsequently inoculated with 1.0 × 10^6^ cfu/mL of *P. aeruginosa* cells. Microplates were incubated at 37 °C, and the MIC was defined as the concentration(s) present in the first optically clear well after 24 h. The effect of combinations of CIP with PEN against *P. aeruginosa* strains was carried out using 96-well microplate assays prepared via doubling dilution of CIP in MHB, followed by subsequent addition of PEN to make a combination checkerboard. Each well was then inoculated with 1.0 × 10^6^ cfu/mL of *P. aeruginosa* cells, and the microplates were incubated at 37 °C. After 24 h, each well was scored for visible growth, and the fractional inhibitory concentration index (FICI) values were calculated for each combination tested. The FICI value was calculated using the equation FICI = Ac/MICa + Bb/MICb, where Ac is the concentration of compound A when combined with compound B; MICA is the MIC of compound A alone; Bc is the concentration of compound B in combination with compound A; and MICb is the MIC of compound B alone. Synergy was defined at the point at which the FICI was ≤0.5. An additive effect was present if the FICI was >1, and antagonism ≥ 4 [36]. Each *P. aeruginosa* strain was tested in duplicate.

### 4.4. Time-Kill Assay

Approximately 1.0 × 10^6^ cfu/mL of *P. aeruginosa* cells were exposed to PBS (control), CIP, and PEN alone or combinations of CIP and PEN in MHB at 37 °C. CIP and PEN, either alone or in combinations, were used at concentrations that represented either MIC_50_ or MIC_25_. Samples were removed for enumeration of viable bacteria after 2, 4, 6, and 24 h of exposure. An initial inoculum was also enumerated as the starting cell number with no exposure to any treatments. Samples were 10-fold serially diluted in MHB prior to plating on Nutrient Agar (NA) plates (Formedium Ltd., Hunstanton, UK). Plates were incubated at 37 °C overnight prior to counting colonies. Susceptibility of each *P. aeruginosa* strain was measured in duplicate, and the mean ± standard error of the mean (SEM) was plotted.

### 4.5. G. mellonella Infection Model

Efficacy of CIP and PEN alone or in combination versus *G. mellonella* larvae infected with the *P. aeruginosa* strains was determined exactly as described previously [35]. *G. mellonella*, at their final instar larval stage, were kept at room temperature in darkness. Larvae weighing within the range of 250 to 350 mg were selected for each experiment to ensure consistency in subsequent drug administration and were used within 1 week of receipt.

Briefly, groups of 15 larvae were infected with an inoculum of 2.5 × 10^3^ cfu/mL of *P. aeruginosa* cells. Treatment with a single dose of CIP or PEN alone or combinations of CIP with PEN were administered 2 h post-infection (p.i). Where stated, in some experiments, a second dose of a treatment was administered at 4 h p.i. All experiments were repeated in duplicate, using larvae from a different batch, and the data from these replicate experiments were pooled to give *n* = 30. Survival data were plotted using the Kaplan–Meier method [37], and comparisons were made between groups by using the log-rank test [38]. In all comparisons with the negative control, it was the uninfected control (rather than the unmanipulated control) that was used, and *p* ≤ 0.05 was considered significant.

The bacterial burden within larvae from treatment groups was measured exactly as described previously [39,40]. Groups of 30 larvae were infected with *P. aeruginosa* cells, using the same inoculum sizes as described above. Treatments of CIP or PEN alone or combinations of CIP with PEN were administered at 2 h p.i. Larvae were incubated in Petri dishes at 37 °C. At 24 h and 96 h p.i, five larvae were randomly selected from each treatment group and surface decontaminated and anesthetized by washing in absolute ethanol. Each larva was then placed in an Eppendorf tube containing 1 mL of sterile PBS and homogenized using a sterile pestle. The bacterial burden from individual caterpillars was then determined by serial dilution of the homogenate in MHB and plating on Pseudomonas Isolation Agar (Sigma–Aldrich Ltd., Dorset, UK). The detection limit for this assay was 100 cfu/mL of larval homogenate.

### 4.6. H33342 Uptake Assay Measuring Efflux Pump Inhibition

The uptake of H33342 to determine the inhibition of efflux was measured as previously described [28]. Briefly, exponential-phase *P. aeruginosa* cells grown in MHB were harvested, washed, and resuspended in PBS containing 1 mM MgSO_4_ and 20 mM glucose at an optical density (600 nm) of 0.4. Then, 100 µL of the bacterial suspension was added to the wells of a 96-well black fluorescence-compatible microplate (Sterilin, Newport, UK). Three conditions were then prepared by adding 50 µL of PBS (with 1 mM MgSO_4_ and 20 mM glucose) alone or with either PEN (256 mg/L) or PAβN (25 mg/L) to each well. PAβN was included as a known efflux-pump inhibitor (EPI) for comparison [41]. Plates were incubated at 37 °C for 15 min before 50 µL of H33342 (2.5 µM) was added to each assay. Fluorescence (excitation, 355 nm; emission, 460 nm) was measured at 37 °C every 2.3 min for 1 h, using a Gemini XPS scanning multimode reader (Molecular Devices, San Jose, CA, USA). All assays were performed in duplicate. The accumulation of H33342 was compared in the absence of an EPI (PBS control) and the presence of a putative EPI (PEN or PAβN). 

### 4.7. NPN Fluorescence Assay Measuring Membrane Permeabilization

Exponential-phase *P. aeruginosa* cells grown in MHB were harvested, washed in 100 mM NaCl and 50 mM sodium phosphate buffer (pH 7.0), and finally resuspended in the same buffer with 0.05% glucose at an optical density (600 nm) of 0.1. Then, 100 µL of the bacterial suspension was added to the wells of a 96-well black fluorescence-compatible microplate (Sterilin, Newport, UK). Three conditions were prepared by adding either 50 µL of PBS, PEN (256 mg/L or 759 µM), or EDTA (100 µM) to each well. EDTA was included as a positive control that is known to permeabilize the outer membrane of Gram-negative bacteria [31]. The, 50 µL of NPN was added to each reaction, and fluorescence (excitation: 322 nm, emission: 322 nm) was measured at 37 °C for 20 min, using a Gemini XPS scanning multimode reader (Molecular Devices, San Jose, CA, USA). Each assay was performed in duplicate. The accumulation of NPN in the absence of an EPI (PBS control) and the presence of PEN or EDTA was compared.

## Figures and Tables

**Table 1:** *P. aeruginosa* strains used.

Strain	Genotype	Phenotype	Reference
NCTC13437	Clinical isolate producing VEB-1; VIM-10 β-lactamases	Resistant to β-lactams and fluoroquinolones by an unknown mechanism	[26]
CR-BJP-VIM	Clinical isolate producing a VIM β-lactamase	Resistant to β-lactams, aminoglycosides, and fluoroquinolones	Clinical isolate
PAM1020	PA01 prototroph	Wild-type parent strain	[24]
PAM1626	Δ*mexAB*-*oprM*::Cm; Δ*mexCD-oprJ*::Gm; Δ*mexEF-oprN*::ΩHg	*mexAB*-*oprM*; *mexCD-oprJ*; and *mexEF-oprN* deleted	[24]
PAM1032	*nalB*-type mutation	*mexAB*-*oprM* overexpressed	[24]
PAM1033	*nfxB*-type mutation	*mexCD-oprJ* overexpressed	[24]
PAM1034	*nfxC*-type mutation	*mexEF-oprN* overexpressed	[24]

**Table 2:** Minimum inhibitory concentrations (MICs) of antimicrobials against *P. aeruginosa* strains determined in MHB after incubation at 37 °C for 24 h. Each experiment was performed at least in duplicate. PEN—pentamidine; CIP—ciprofloxacin.

		MIC (mg/L)	
Strain	Phenotype	PEN	CIP
NCTC13437	Resistant to β-lactams and fluoroquinolones	256	32
CR-BJP-VIM	Resistant to β-lactams, aminoglycosides, and fluoroquinolones	256–512	32
PAM1020	Isogenic parent strain of efflux pump mutants	256	0.0625
PAM1032	Overexpression of MexAB-OprM	256	0.5–1
PAM1033	Overexpression of MexCD-OprJ	256–512	1
PAM1034	Overexpression of MexEF-OprN	256–512	1
PAM1626	Triple deletion of MexAB-OprM, MexCD-OprJ, and MexEF-OprN	16	0.0078

## Data Availability

Data can be made available by the corresponding author upon request.
